# Cluster of Influenza A(H5) Cases Associated with Poultry Exposure at Two Facilities — Colorado, July 2024

**DOI:** 10.15585/mmwr.mm7334a1

**Published:** 2024-08-29

**Authors:** Cara C. Drehoff, Elizabeth B. White, Aaron M. Frutos, Ginger Stringer, Alexis Burakoff, Nicole Comstock, Alicia Cronquist, Nisha Alden, Isaac Armistead, Allison Kohnen, Radhika Ratnabalasuriar, Emily A. Travanty, Shannon R. Matzinger, Alexandria Rossheim, Aleigha Wellbrock, H. Pamela Pagano, Dennis Wang, Jordan Singleton, Rebekah A. Sutter, C. Todd Davis, Krista Kniss, Sascha Ellington, Marie K. Kirby, Carrie Reed, Rachel Herlihy, H5N1 Field Investigation Team, H5N1 Field Investigation Team, Robyn Weber, Leovi Madera, Megan Vizina, Brene Belew-Ladue, Hannah Padda, Angiezel Merced-Morales, Ann Carpenter, Grace E. Marx, Lizette O. Durand, Scott Brueck

**Affiliations:** ^1^Colorado Department of Public Health & Environment; ^2^Epidemic Intelligence Service, CDC; ^3^Influenza Division, National Center for Immunization and Respiratory Diseases, CDC; ^4^Coronavirus and Other Respiratory Viruses Division, National Center for Immunization and Respiratory Diseases, CDC; ^5^Divison of Vector-Borne Diseases, National Center for Emerging and Zoonotic Infectious Diseases, CDC.; Colorado Department of Public Health & Environment; Colorado Department of Public Health & Environment; Colorado Department of Public Health & Environment; Colorado Department of Public Health & Environment; 2024 Influenza A (H5N1) Response, CDC; 2024 Influenza A (H5N1) Response, CDC; 2024 Influenza A (H5N1) Response, CDC; 2024 Influenza A (H5N1) Response, CDC; 2024 Influenza A (H5N1) Response, CDC; 2024 Influenza A (H5N1) Response, CDC

**Summary**

**What is already known about this topic?**

Humans who have contact with influenza A(H5N1) virus–infected cattle or poultry can become infected.

**What is added by this report?**

The first known cluster of human influenza A(H5) cases in the United States associated with poultry exposure occurred in Colorado; 109 (16.4%) of 663 workers performing poultry depopulation reported symptoms and received testing, and nine (8.3%) of the workers who received testing for influenza A(H5) received a positive result. All nine cases were associated with mild illness, with conjunctivitis as the most common symptom.

**What are the implications for public health practice?**

As the prevalence of highly pathogenic avian influenza A(H5N1) virus clade 2.3.4.4b genotype B3.13 increases, U.S. public health agencies should prepare to rapidly investigate and respond to illness in agricultural workers, including workers with limited access to health care.

## Abstract

Persons who work in close contact with dairy cattle and poultry that are infected with highly pathogenic avian influenza (HPAI) A(H5N1) virus are at increased risk for infection. In July 2024, the Colorado Department of Public Health & Environment responded to two poultry facilities with HPAI A(H5N1) virus detections in poultry. Across the two facilities, 663 workers assisting with poultry depopulation (i.e., euthanasia) received screening for illness; 109 (16.4%) reported symptoms and consented to testing. Among those who received testing, nine (8.3%) received a positive influenza A(H5) virus test result, and 19 (17.4%) received a positive SARS-CoV-2 test result. All nine workers who received positive influenza A(H5) test results had conjunctivitis, experienced mild illness, and received oseltamivir. This poultry exposure–associated cluster of human cases of influenza A(H5) is the first reported in the United States. The identification of these cases highlights the ongoing risk to persons who work in close contact with infected animals. Early response to each facility using multidisciplinary, multilingual teams facilitated case-finding, worker screening, and treatment. As the prevalence of HPAI A(H5N1) virus clade 2.3.4.4b genotype B3.13 increases, U.S. public health agencies should prepare to rapidly investigate and respond to illness in agricultural workers, including workers with limited access to health care. 

## Investigation and Results

### Public Health Notification and Response

On July 8, 2024, poultry in a commercial egg-layer operation in northeast Colorado (facility A)* were confirmed to have highly pathogenic avian influenza (HPAI) A(H5N1).^†^ Facility A hired approximately 250 contract workers to conduct depopulation (i.e., euthanasia) of all poultry on the premises, which began on July 9. On July 11, the Colorado Department of Public Health & Environment (CDPHE) and Colorado Department of Agriculture were notified of several ill workers. Based on potential exposure and symptoms consistent with influenza A(H5N1) virus infection, a field team was mobilized to conduct testing among symptomatic workers and offer them empiric treatment with the influenza neuraminidase inhibitor oseltamivir (75 mg twice daily for 5 days). Seven workers reported symptoms and received testing on July 11, and 45 symptomatic workers received testing on July 12; all received oseltamivir. To ensure an adequate supply of the recommended personal protective equipment (PPE) for exposed workers (*1*), CDPHE delivered goggles, N95^§^ filtering facepiece respirators (FFRs), and nitrile gloves to facility A on July 12. On July 13, a small team returned to determine PPE-use practices during work activities.

Because many workers had symptoms, including several who received presumptive positive test results for influenza A(H5),^¶^ and because observed PPE compliance was low, CDPHE distributed oseltamivir to all workers as postexposure prophylaxis (PEP),** irrespective of symptoms. On July 15 and 16, an on-site team conducted symptom screening, testing for symptomatic workers, and distribution of oseltamivir; 13 additional workers with symptoms received testing and empiric treatment, and 219 workers received a 10-day course of oseltamivir PEP.^††^ CDPHE returned to facility A on July 23, and identified no additional workers with symptoms.

On July 14, 2024, CDPHE was notified that poultry at facility B, located in the same county as facility A, had a nonnegative test result^§§^ for influenza A(H5). CDPHE delivered goggles and N95 FFRs to facility B on July 15. Facility B commenced poultry depopulation on July 15, with approximately 400 contract workers participating. The facility initially reported no illness among workers and high PPE compliance. Therefore, oseltamivir PEP was not offered. Instead, CDPHE established routine screening and offered testing and empiric oseltamivir treatment (75 mg twice daily for 5 days) during six visits^¶¶^ to 44 workers experiencing symptoms.

Between CDPHE site visits, staff member team leads at facilities A and B conducted screening among workers before shifts based on guidance from CDPHE. Facility A identified no additional symptomatic workers after July 16. Facility B reported two symptomatic workers during facility-led screening; both workers declined testing and empiric oseltamivir treatment. As depopulation activities concluded, CDPHE visited both facilities to distribute cards providing information in English and Spanish about symptoms of avian influenza A virus infection in humans, where to seek care if workers became ill, and information for health care providers regarding workers’ exposure to H5N1-infected poultry.

### Screening and Testing Among Workers

Workers conducting poultry depopulation, carcass removal, and disposal were asked if they were feeling ill. Those with self-reported symptoms were asked to complete a brief questionnaire including information on exposures, symptom onset, specific symptoms,*** and PPE use.^†††^ Nasopharyngeal swabs and conjunctival swab specimens were collected from workers reporting symptoms; swab specimens were tested for influenza A and A(H5) virus at the CDPHE laboratory. Specimens testing negative for influenza A and A(H5) virus were tested for SARS-CoV-2.^§§§^ Specimens testing presumptively positive for influenza A(H5) virus or with inconclusive results were sent to CDC for confirmatory testing. This activity was reviewed by CDC, deemed not research, and was conducted consistent with applicable federal law and CDC policy.^¶¶¶^

CDPHE screened 663 workers for symptoms during July 11–July 26, 2024. The median age of workers was 30 years (range = 15–56 years), and most spoke only Spanish. At facility A, 65 (25%) of 265 workers who received screening reported symptoms and received testing, and six (9%) of 65 (2.3% of all workers) received a positive influenza A(H5) test result (Table 1). At facility B, 44 (11%) of 398 workers who received screening reported symptoms and received testing, and three (7%) of these 44 workers (0.8% of all workers) received a positive influenza A(H5) test result. Among those who received a negative influenza A and A(H5) test result, one worker at facility A and 18 at facility B received a positive SARS-CoV-2 test result. Symptom onset date was known for 25 (38%) of 65 workers at facility A and 39 (89%) of 44 workers at facility B (Figure).

**TABLE 1:** Influenza A(H5) test result, age, and use of personal protective equipment among symptomatic workers conducting depopulation in two poultry facilities — Colorado, July 2024

Characteristic	No. (%) of symptomatic workers	
	Facility A N = 65	Facility B N = 44
**Influenza A(H5) cases, by PCR**	**6 (9)**	**3 (7)**
**Median age, yrs (IQR)**	**35 (27–41)**	**28 (23–35)**
Self-reported PPE use*		
Eye protection	28 (43)	38 (86)
Mask	32 (49)	44 (100)
Coveralls	23 (35)	41 (93)
Gloves	20 (31)	43 (98)
Boots or boot covers	12 (18)	41 (93)
Head cover	20 (31)	34 (77)

**Abbreviations**: FFR = filtering facepiece respirator; PCR = polymerase chain reaction; PPE = personal protective equipment.

* PPE provided by producers varied but included N95 and KN95 FFRs, Tyvek suits, boot covers, nitrile gloves, safety glasses, and goggles. Many workers owned shoes designated for work activities that remained at the facility. The Colorado Department of Public Health & Environment provided goggles, nitrile gloves, and N95 FFRs.

**FIGURE:**
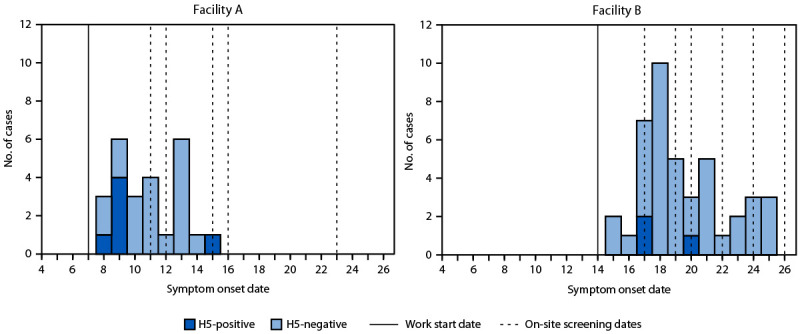
Work start date,* on-site screening dates, and known symptom onset dates^†^ for symptomatic workers who received testing results for influenza A(H5), by poultry facility — Colorado, July 2024 **Abbreviation:** H-5 = influenza A(H5). * Work start date was defined as the date when depopulation or disposal of sick poultry began at the facility. ^†^ Symptom onset was known for 25 (38%) of 65 symptomatic workers at facility A and 39 (89%) of 44 symptomatic workers at facility B.

### PPE Use Among Workers Who Were Symptomatic

Self-reported PPE use among workers who were symptomatic varied by facility. At facility A, workers reported lowest usage for boots or boot covers (18%) and highest usage for masks (49%). At facility B, workers reported lowest usage for head covers (77%) and highest usage for masks (100%).

### Clinical Description of Human Influenza A(H5) Cases

All nine workers who received positive influenza A(H5) test results completed the questionnaire at the time of testing, and eight were reached for detailed interviews after receipt of their positive test result. Five cases occurred among women and four among men. The median age was 32 years (range = 18–56 years). Two patients had diabetes, one had asthma, and one was a longtime smoker. All reported direct contact with sick or dead poultry during depopulation and carcass disposal activities. Symptom onset occurred a median of 1 day after initial occupational exposure (range = 1–8 days),**** and symptomatic workers received testing a median of 2 days after symptom onset (range = 0–3 days). All nine patients reported conjunctivitis, seven reported eye tearing, and six reported subjective fever or chills. All patients reporting subjective fever or chills worked at facility A. Respiratory symptoms such as sore throat, cough, and shortness of breath were less frequently reported (Table 2). All patients received oseltamivir treatment. Symptoms resolved for seven patients a median of 4 days after onset (range = 1–8 days). Two patients interviewed 2 days after symptom onset reported ongoing or improving conjunctivitis; however, these persons were not able to be interviewed again. No hospitalizations or deaths occurred; one patient sought outpatient medical care for conjunctivitis on the day of symptom onset. Four patients remained symptomatic and were retested 1–5 days after receipt of their initial positive test result; none received a positive follow-up test result. Among the nine workers who received a positive test result for influenza A(H5), both nasopharyngeal and conjunctival swabs were positive for three, only the conjunctival swab was positive for five, and only the nasopharyngeal swab was positive for one. Virus was successfully isolated from specimens from five infected workers, codon complete genomes were successfully sequenced for four cases, and six of eight gene segments were successfully sequenced for one, identifying the viruses as clade 2.3.4.4b genotype B3.13.

**TABLE 2:** Reported symptoms among workers conducting depopulation who received screening by Colorado Department of Public Health & Environment, by influenza A(H5) test result and poultry facility — Colorado, July 2024

Symptom	Influenza A(H5) test result, no. (%)					
	A(H5)-positive			A(H5)-negative		
	Overall N = 9	Facility A n = 6	Facility B n = 3	Overall N = 100	Facility A n = 59	Facility B n = 41
Red eyes/Conjunctivitis	9 (100)	6 (100)	3 (100)	66 (66)	42 (71)	24 (59)
Eye tearing	7 (78)	5 (83)	2 (67)	51 (51)	36 (61)	15 (37)
Fever or chills	6 (67)	6 (100)	0 (—)	33 (33)	13 (22)	20 (49)
Cough	3 (33)	3 (50)	0 (—)	38 (38)	13 (22)	25 (61)
Sore throat	4 (44)	4 (67)	0 (—)	62 (62)	32 (54)	30 (73)
Runny or stuffy nose	2 (22)	2 (33)	0 (—)	41 (41)	15 (25)	26 (63)
Sneezing	1 (11)	1 (17)	0 (—)	15 (15)	5 (8)	10 (24)
Difficulty breathing	1 (11)	1 (17)	0 (—)	7 (7)	1 (2)	6 (15)
Shortness of breath	3 (33)	3 (50)	0 (—)	10 (10)	1 (2)	9 (22)
Fatigue	1 (11)	1 (17)	0 (—)	16 (16)	2 (3)	14 (34)
Rash	0 (—)	0 (—)	0 (—)	0 (—)	0 (—)	0 (—)
Body aches	5 (56)	5 (83)	0 (—)	28 (28)	5 (8)	23 (56)
Headaches	5 (56)	5 (83)	0 (—)	38 (38)	12 (20)	26 (63)
Nausea	3 (33)	2 (33)	1 (33)	16 (16)	5 (8)	11 (27)
Vomiting	1 (11)	1 (17)	0 (—)	9 (9)	1 (2)	8 (20)
Diarrhea	1 (11)	1 (17)	0 (—)	12 (12)	6 (10)	6 (15)
Seizures	0 (—)	0 (—)	0 (—)	0 (—)	0 (—)	0 (—)

## Discussion

Before this outbreak, five human cases of influenza A(H5) had been reported in the United States: one in 2022 in Colorado associated with poultry exposure^††††^ and four among dairy workers reported during April–July 2024 associated with clade 2.3.4.4b genotype B3.13 circulating in dairy cattle (*2*,*3*). HPAI A(H5N1) has been detected both in dairy cattle herds^§§§§^ and poultry flocks in Colorado this year.^¶¶¶¶^ This report describes the first cluster of U.S. cases associated with a common source of occupational exposure to poultry. The identification of nine cases across two poultry facilities highlights the ongoing risk to persons who work in close contact with infected animals.

Epidemiologic and clinical characteristics of cases in this cluster were similar to those in U.S. human cases of influenza A(H5) associated with exposure to dairy cattle (*2*,*4*). All infected workers had occupational exposure to sick or dead poultry, and all reported mostly mild symptoms. However, influenza A(H5N1) virus is known to result in a broad spectrum of illness among humans, including severe disease and death (*5*), underscoring the importance of prompt investigation and treatment of potential human cases (*6*). Although environmental contamination (e.g., nasal or ocular carriage of noninfectious viral particles) cannot be ruled out in this cluster, evidence suggests that many of these cases represent actual infection. Four of nine cases occurred in persons who received testing as they arrived at work in the morning, before exposure to environmental or occupational contaminants occurred on the day of testing.***** In addition, all nine infected workers reported symptoms of conjunctivitis and received testing within 3 days of symptom onset; conjunctivitis has been observed in previous cases with occupational exposure to HPAI-infected poultry (*7*). Influenza A(H5N1) virus was also isolated, and full-length gene segments were sequenced from clinical specimens collected from five patients.

Poultry depopulation activities and their attendant environments are associated with high potential for viral exposure at affected facilities. In addition to handling and disposing of dead birds, the predominant depopulation method used at both facilities also involved handling each live bird,^†††††^ which increased exposure and the risk for displacement of or damage to PPE, especially in these cage-free facilities where birds roam free and must be physically caught. Agricultural worker health and safety should be prioritized by employers through the use of engineering, administrative, and PPE controls. Challenges were reported and observed in the acquisition, provision, and training in the use of proper PPE for a large number of workers who were urgently hired to depopulate poultry. Self-reported PPE use was low for certain components, observations revealed some inconsistent or improper PPE use, and extreme heat made compliance difficult. However, cases were also identified in facility B where frequency of PPE use was higher, but still <100%.

This cluster of influenza A(H5) cases in a predominantly Spanish-speaking migrant workforce highlights the importance of a public health response that prioritizes health equity. Multilingual teams including Spanish speakers were fundamental to building trust and conducting postexposure screening and testing and providing treatment. The robust public health response by CDPHE, including on-site screening and timely testing of symptomatic workers, increased access to care and likely optimized case-finding. Testing was also critical to identifying cases from a larger cohort of symptomatic persons working in close contact in an enclosed environment, which can facilitate spread of respiratory pathogens such as SARS-CoV-2, and environmental respiratory irritation was likely (*8*).

### Implications for Public Health Practice

These findings suggest that poultry workers who are exposed to enclosed environments with birds infected with HPAI A(H5N1) virus are at increased risk for infection. Given the continued circulation of this virus in the United States, public health agencies should proactively prepare for additional human cases in both dairy and poultry facilities. This preparation should include distributing PPE; training public health field teams on proper PPE use; determining the logistics of large-scale screening, specimen collection, and laboratory testing to distinguish influenza A(H5) virus from seasonal respiratory viruses; acquiring oseltamivir; and developing standardized protocols for empiric treatment or PEP with oseltamivir. In addition, response preparation should include the cultural and language needs of the agricultural workforce in the jurisdiction. A One Health^§§§§§^ approach that takes into consideration human, animal, and environmental health is also required for a timely and coordinated response, including collaboration with industry, labor, and regulatory agriculture partners.

## Footnotes

* Facility A was a large commercial operation with 1.8 million egg-laying poultry. Facility B was a large commercial operation with 1.3 million egg-laying poultry. Facility B was located in the same county as facility A, but the two facilities had no connection to each other.

† Confirmatory testing was conducted by the National Veterinary Services Laboratory (NVSL), and the virus was later identified as clade 2.3.4.4b genotype B3.13. Facility A received a confirmatory laboratory result by NVSL on July 8; facility B received confirmation by NVSL on July 16.

§ N95 is a certification mark of the U.S. Department of Health and Human Services registered in the United States and several international jurisdictions.

¶ Samples were considered presumptively positive until testing at CDC confirmed the positive result.

**
https://www.cdc.gov/bird-flu/spotlights/hpai-health-recommendations.html

†† On July 15–16, 188 workers received a 10-day course of oseltamivir PEP. Thirty-one workers who received testing July 11–12, initially received a 5-day oseltamivir course for empiric treatment, and received negative test results were offered an additional 5-day supply of oseltamivir for the complete 10-day PEP course. The 10-day course was offered because of ongoing occupational exposure.

§§ A nonnegative test result refers to an initial detection in poultry, as opposed to detection of a human case.

¶¶ Worker-reported illness and testing participation at facility B site visits: July 17 (one), July 19 (18), July 20 (five), July 22 (six), July 24 (four), and July 26 (10).

*** Symptoms included red eyes, eye tearing, subjective fever or chills, cough, sore throat, runny or stuffy nose, sneezing, difficulty breathing, shortness of breath, fatigue or feeling very tired, muscle or body aches, headaches, nausea, vomiting, diarrhea, seizures, or rash.

††† The survey asked if workers typically used any of the following during work activities: eye protection, mask, coveralls, gloves, boots or boot covers, or head covering. PPE provided by producers varied but included N95 and KN95 FFRs, Tyvek suits, booties, nitrile gloves, safety glasses, and goggles. CDPHE also provided goggles, nitrile gloves, and N95 FFRs. The survey did not distinguish among masks, N95 respirators, or other types of face coverings.

§§§ Samples were tested for influenza A(H5) via reverse transcription polymerase chain reaction on the CDC Influenza A/H5 Subtyping Diagnostic assay. Samples that tested negative for influenza A(H5) were tested for SARS-CoV-2 on the CDC Influenza SARS-CoV-2 (Flu SC2) Multiplex assay.

¶¶¶ 45 C.F.R. part 46.102(l)(2), 21 C.F.R. part 56; 42 U.S.C. Sect. 241(d); 5 U.S.C. Sect. 552a; 44 U.S.C. Sect. 3501 et seq.

**** Date of initial exposure was defined as the date when depopulation or disposal of sick poultry began, or the person’s first day working at the facility, whichever was later.

††††
https://emergency.cdc.gov/han/2022/han00464.asp

§§§§ https://www.aphis.usda.gov/livestock-poultry-disease/avian/avian-influenza/hpai-detections/hpai-confirmed-cases-livestock (Accessed August 2, 2024).

¶¶¶¶ https://www.aphis.usda.gov/livestock-poultry-disease/avian/avian-influenza/hpai-detections/commercial-backyard-flocks (Accessed August 2, 2024).

***** These workers received testing as they reported for work on a given day; however, they had worked on previous days, when they were presumably exposed.

††††† Containerized gassing with carbon dioxide was determined as the preferred depopulation method in consultation with the Colorado Department of Agriculture and U.S. Department of Agriculture in accordance with American Veterinary Medical Association recommendations. Facility A used this method in 100% of bird houses, and facility B used it in approximately 55% of bird houses. https://www.avma.org/resources-tools/avma-policies/avma-guidelines-depopulation-animals

§§§§§
https://www.cdc.gov/one-health/
